# Cell line selection using the Duetz Microflask system

**DOI:** 10.1186/1753-6561-5-S8-P15

**Published:** 2011-11-22

**Authors:** Steve R C Warr, Sharon L White, Yuen-Ting Chim, Jai Patel, Hella Bosteels

**Affiliations:** 1 GlaxoSmithKline, Stevenage, Hertfordshire, SG1 2NY, UK

## Introduction

The identification of a small number of monoclonal antibody (mAb) producing candidate cell lines from the large number of clones generated post transfection is one of the bottlenecks of cell line development. Clone numbers are reduced significantly during initial medium exchange and static scale up stages but significant numbers can still progress to evaluation in shaking cultures. This is often carried out in shake flasks where the number of clones that can be evaluated may be restricted due to resource limitations.

The Duetz Microflask system is a microtitre plate based system which uses ‘Sandwich Covers’ to convert the individual wells of 24 well plates into individual ‘mini reactors’. The ‘Sandwich Covers’ consist of a stainless steel cover, a 0.2µm filter, microfibre inlays and a flexible silicone sealing layer to ensure adequate oxygen transfer rates to individual wells.

This work describes the use of the Duetz Microflask system to evaluate cell lines in culture prior to scale up to production shake flasks. We have compared the growth and rank order of performance for a number of cell lines in the Duetz Microflask system with data obtained from shake flasks and demonstrated this system can be used successfully to identify candidate cell lines for further progression.

A series of preliminary experiments was completed to determine suitable volumes and operating conditions to minimise evaporation. Unless otherwise stated these were: Fill volume – 1.5mL, Shaker speed – 200rpm, Humidity – 80%.

## Cell line evaluation

After initial transfection a typical cell line development process would involve plating cell lines to multiwell plates followed by several static media exchange, scale up and selection steps. These are used to reduce the number of cell lines carried forward to larger scale static T-flasks and eventually to shake flasks for further evaluation and selection. Although the multiwell plate steps are amenable to automation the manual handling aspects of culturing multiple cell lines in shake flasks can be resource limiting and therefore the ability to improve cell line selection using shaking multiwell plates offers significant time line and resource advantages.

## Complex medium process

The performance of a series of 28 mAb producing clones in hydrolysate containing media in Duetz Microflasks was compared with standard shake flask evaluation data.

The rank order of titre performance of these clones in ‘Early’ Duetz Microflasks (ie inoculated before repeated shake flask subculture) was compared to that in shake flasks inoculated after the cultures had been subcultured for 6 passages. There were some differences in the rank orders resulting in a correlation R^2^ = 0.46. However this improved significantly (R^2^ = 0.92) when a further set of Duetz Microflasks (‘Late’ Duetz) was run in parallel to the flasks.

This data indicates that although Duetz Microflasks can be used at an early screening stage to identify high producing clones the correlation with subsequent performance is improved after the cell lines are adapted to shaking growth conditions (ie after repeated subculture in shake flasks).

**Figure 1:**
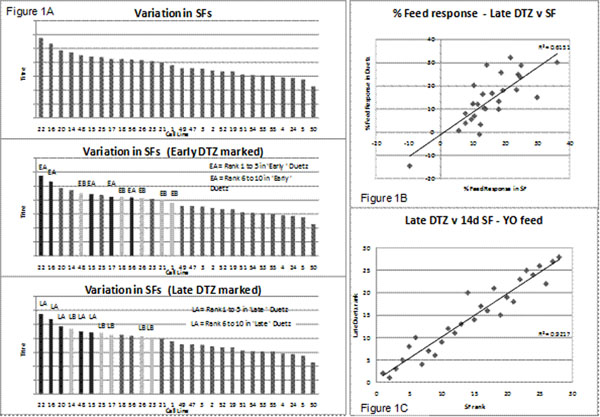
Titre rank order of clones in batch shake flasks compared with top clones in batch ‘Early’ ‘Late’ Duetz Microflasks (Figure 1A). Correlation of the % feed response (Figure 1B) and titre rank order (Figure 1C) of clones in a fed batch process in shake flasks and ‘Late’ Duetz Microflasks is also shown.

Figure 1A shows that the top 8 cell lines identified in a ‘Late’ Duetz screen were the same as the top 8 lines identified in shake flasks. In contrast only 5 of the top 8 lines in the ‘Early’ Duetz screen were in the top 8 lines in shake flasks. Therefore the likelihood of discarding a high producing line is increased the earlier the Duetz screening is carried out.

Similar results were obtained using a hydrolysate feed fed batch process in which the titre rank order and the response to feed in Duetz Microflasks was similar to that in shake flasks. Thus Figure 1B shows good correlation between the effect of the feed (% increase in titre) on cell lines in shake flasks and in ‘Late’ Duetz Microflasks and Figure 1C shows the correlation between the (titre) rank order in the two systems.

## Chemically defined medium process

A similar experiment was carried out with a different mAb producing CHO cell line in a chemically defined fed batch process. The standard (bioreactor and SF model) process involves a series of feeds during the fermentation. This multiple feed strategy was found to be impractical at the Microflask scale and so a compromise ‘single feed’ model was used in Duetz Microflasks. The correlation of rank order between ‘Early’ Duetz Microflasks and shake flasks was poor (R^2^ = 0.34) but was significantly improved after Duetz Microflasks were inoculated with cells previously subcultured in shake flasks (R^2^ = 0.73).

## Discussion

This data has demonstrated that, although there are some limitations around the adaptation of cell lines to shaking culture, Duetz Microflask screening can be used successfully to reduce the number of cell lines carried forward in the cell line selection process to shake flask or bioreactor screening.

Although correlation with subsequent shake flasks is improved after repeated subculture (to allow complete adaptation to shaking) this data has also demonstrated that screening in Duetz Microflasks inoculated directly from static cultures or after minimal shake flask subculturing can be used to differentiate between cell lines that are high and low performers in conventional screening systems. We have used this system to reduce the number of cell lines carried forward to shake flask screening by approximately 70%.

The simplicity of this system combined with the standard multiwell plate footprint facilitates automation and potentially enables large numbers of cell lines to be screened simultaneously and we are currently incorporating this system into our automated cell line generation process.

